# Publisher Correction: Contemporaneous 3D characterization of acute and chronic myocardial I/R injury and response

**DOI:** 10.1038/s41467-019-10804-x

**Published:** 2019-06-19

**Authors:** Simon F. Merz, Sebastian Korste, Lea Bornemann, Lars Michel, Pia Stock, Anthony Squire, Camille Soun, Daniel R. Engel, Julia Detzer, Holger Lörchner, Dirk M. Hermann, Markus Kamler, Joachim Klode, Ulrike B. Hendgen-Cotta, Tienush Rassaf, Matthias Gunzer, Matthias Totzeck

**Affiliations:** 1 Institute for Experimental Immunology and Imaging, University Hospital, University Duisburg-Essen, 45147 Essen, Germany; 2 Department of Dermatology, Venerology and Allergology, University Hospital Essen, 45147 Essen, Germany; 3 Department of Cardiology and Vascular Medicine, University Hospital Essen, 45147 Essen, Germany; 4 Dept. of Cardiac Development and Remodelling, Max Planck Institute for Heart and Lung Research, 61231 Bad Nauheim, Germany; 5 German Centre for Cardiovascular Research (DZHK), Partner site Rhine-Main, Frankfurt am Main, Germany; 6 Department of Neurology, University Hospital Essen, 45147 Essen, Germany; 7 Department of Thoracic and Cardiovascular Surgery, University Hospital Essen, 45147 Essen, Germany

**Keywords:** Cell death and immune response, Myocardial infarction

Correction to: *Nature Communications* 10.1038/s41467-019-10338-2, published online 24 May 2019.

The original version of this Article contained errors in the author affiliations.

The affiliations of Lea Bornemann, Anthony Squire, Camille Soun, Daniel R. Engel and Matthias Gunzer with ‘Institute for Experimental Immunology and Imaging, University Hospital Essen, 45147, Essen, Germany’ were inadvertently omitted.

Additionally, Matthias Gunzer was incorrectly associated with ‘Department of Dermatology, Venerology and Allergology, University Hospital Essen, 45147 Essen, Germany.’

This has now been corrected in both the PDF and HTML versions of the Article.

