# Supplementary material for: Contemporaneous 3D characterization of acute and chronic myocardial I/R injury and response
Source: Nat Commun. 2019 May 24;10:2312. doi: 10.1038/s41467-019-10338-2 (PMC6534576; doi:10.1038/s41467-019-10338-2)
Supplement: Supplementary file 1 — Supplementary Information [file 41467_2019_10338_MOESM1_ESM.pdf]

Supplementary material relating to

Contemporaneous 3D characterization of acute and chronic myocardial I/R injury and response

Simon F. Merz*[1,2], Sebastian Korste*[3], Lea Bornemann*[1], Lars Michel[3], Pia Stock[3], Anthony Squire[1], Camille Soun[1], Daniel R. Engel[1], Julia Detzer[4], Holger Lörchner[4,5], Dirk M. Hermann[6], Markus Kamler[7], Joachim Klode[2], Ulrike Hendgen-Cotta[3], Tienush Rassaf[3], Matthias Gunzer[#1] and Matthias Totzeck[#3]

**These authors contributed equally*
#These authors jointly supervised this work

[1]Institute for Experimental Immunology and Imaging, University Hospital Essen, 45147, Essen, Germany

[2]Department of Dermatology, Venerology and Allergology, University Hospital Essen, 45147, Essen, Germany

[3]Department of Cardiology and Vascular Medicine, University Hospital Essen, 45147, Essen, Germany

[4]Max Planck Institute for Heart and Lung Research, Dept. of Cardiac Development and Remodelling, 61231, Bad Nauheim, Germany

[5]German Centre for Cardiovascular Research (DZHK), Partner site Rhine-Main, Frankfurt am Main, Germany

[6]Department of Neurology, University Hospital Essen, 45147, Essen, Germany

[7]Department of Thoracic and Cardiovascular Surgery, University Hospital Essen, 45147, Essen, Germany

Corresponding author:
Dr. Matthias Totzeck
Department for Cardiology and Vascular Medicine
West German Heart and Vascular Center
Medical Faculty, University Hospital Essen
University Duisburg-Essen
Hufelandstraße 55, 45147 Essen, Germany
Phone:+49 201 723 4818
Fax: +49 201 723 5401
Matthias.Totzeck@uk-essen.de

Prof. Dr. Matthias Gunzer
Institute for Experimental Immunology and Imaging
Medical Faculty, University Hospital Essen
University Duisburg-Essen
Hufelandstraße 55, 45147 Essen, Germany
Phone:+49 201 183 6640
Fax: +49 201 183 6642
Matthias.Gunzer@uni-due.de

Supplementary Note 1

Alternative bleaching protocols

We tested the efficiency of BALANCE against Sudan Black bleaching and heme elution, both established protocols for reducing tissue autofluorescence[1]. Sudan Black bleaching was conducted before dehydration of the sample. However, incubation with Sudan Black left the heart tissue stained black, preventing laser penetration into the tissue for more than a few micrometers (Supplementary Figure 1 b). For heme elution, we incorporated CUBIC-1 reagent[2] incubation steps before the original ethyl cinnamate (ECi) protocol's[3] dehydration. This extended the time needed until the imaging process by 2 d (compared to the BALANCE protocol). We achieved autofluorescence homogenization of some parts of the heart, while we still found several spots of high background signal (Supplementary Figure 1 c).

Supplementary Note 2

Endogenous fluorophores

We used the Catchup mouse model[4] expressing tdTomato in neutrophils to benchmark our i.v.-mediated Ly-6G staining in terms of cellular labeling efficiency. Clearing an infarcted murine Catchup heart with ethyl cinnamate (without peroxide treatment) allowed for visualization of both, i.v.-mediated Ly-6G labeling and tdTomato signal (Supplementary Figure 2 c and d)[3]. Here, we found that all tdTomato positive cells in the tissue were also positive for Ly 6G labeling, strengthening the applicability of this antibody delivery route for immune cells and surface markers. However, the signal-to-noise ratio of tdTomato was low, with signal intensities matching muscle autofluorescence. Using BALANCE, we lowered the tissue autofluorescence in order to enhance sample clarity and signal homogeneity of synthetic dyes. However, hereby endogenously expressed tdTomato was quenched as well (Supplementary Figure 2 b). Additionally, when using the CUBIC protocol[2], we were able to visualize the endogenous fluorescence, but observed less sample clarity and lost the artificial Ly-6G staining completely (Supplementary Figure 2 a; this caveat is described in the original paper and can be potentially circumvented by further establishment).

Supplementary Table 1 Comparison of alternative bleaching approaches with the BALANCE protocol. No bleaching[3], Sudan Black bleaching[1] and heme elution[1,2] were conducted as previously published.

	Autofluorescence homogenization	Sample clarity	Endogenous fluorophore bleaching	Fluorescent labeling
**No bleaching[3]**	no homogenization in 488 and 561	grid lines hardly visible	no	preserved
**Sudan Black[1]**	no homogenization in 488, 561 and 647	grid lines not visible	N.A.	preserved
**Heme elution[1,2]**	homogenous in all channels; autofluorescent spots	grid lines visible; brown color	no	reduced
**BALANCE**	homogenous in all channels	grid lines visible; reduced color	yes	preserved

Supplementary Table 2 Comparison of established clearing methods with the BALANCE protocol (shrinkage is mean ± s.d.). Evaluation of CUBIC[5], CLARITY[5], iDISCO[6] and SWITCH[6] as previously published.

	Macroscopic sample clarity	Autofluorescence homogenization	Endogenous fluorophores preserved	Tissue alteration	Sample integrity	Incubation time	Characteristics
**ECi**	Moderate - brown	No	Yes	Slight shrinkage (~20%)	Stiff	1 d	Non-toxic
**BALANCE**	Clear - light yellow	Yes	No	Slight shrinkage (18% ± 2)	Stiff	1.5 d	Non-toxic
**CUBIC[5]**	Moderate – light brown	Not tested	Yes	Expansion	Spongy	14 d	Non-toxic
**CLARITY[5]**	Moderate – light brown	Not tested	Yes	Expansion	Spongy	10 d	Toxic
**iDISCO[6]**	Clear – light brown	Yes	Yes	Shrinkage	Stiff	Hours – days	Toxic
**SWITCH[6]**	Clear – light yellow	Yes	No	Slight expansion	Not tested	Days	Toxic

Supplementary Table 3 Comparison of assessed parameters, shortcomings, time-expense and difficulty of available techniques to evaluate myocardial ischemia/reperfusion (I/R) injury and associated parameters. (2D – two-dimensional, AAR – area at risk, d – days, h – hours, LSFM – light sheet fluorescence microscopy, TTC – triphenyl tetrazolium chloride; asterisk - protocol time depends on automation processes)

Methods	Parameters assessed	Shortcomings	Total time until results	Hands-on time	Analysis time	Difficulty
**TTC-planimetry**	Myocardial infarction parameters:<br>Infarct size, AAR, remote zone (with blue dye),<br>In pseudo-3D	No single cell resolution	2.5 h	1 h	0.25 h	Simple, parameters vary depending e.g. on operator and camera settings
**Flow cytometry**	Immune cell quantification and subset analysis	No precise assessment of I/R areas, no cell localization context	3.5 h	1.5 h	0.5 h	Simple to advanced analysis, depending on staining panel
**(Immuno-) Histology**	Infarct size, immune cell localization and quantification	Limited 3D spatial information due to thin 2D slices (<10µm);<br>3D information in sequential slices possible, but high time and cost expense and computational power involved	3-7 d	4 h	1-2 h, depending on level of analysis	Simple (H&E) to advanced (multicolor sequential immunohisto-chemistry)
**BALANCE/ LSFM**	Multiplexed infarction parameters (vascular damage, AAR and remote zone) with target cells in 3D; Identification of regions of interest for further processing (e.g. immuno-histology)	Need for bleaching, due to whole organ imaging;<br>target cell detection restricted to surface markers (i.v.-mediated staining), limited multiplexing.<br>3D rendering software and computational power required	1.5–4 d*	2.5 h	1.5 h for basic I/R injury parameters and cell segmentation; segment model or distance analysis more time consuming	Simple (infarction parameters and 3D immune cell localization) to advanced, depending on staining panel and post-processing

Supplementary Figure 1

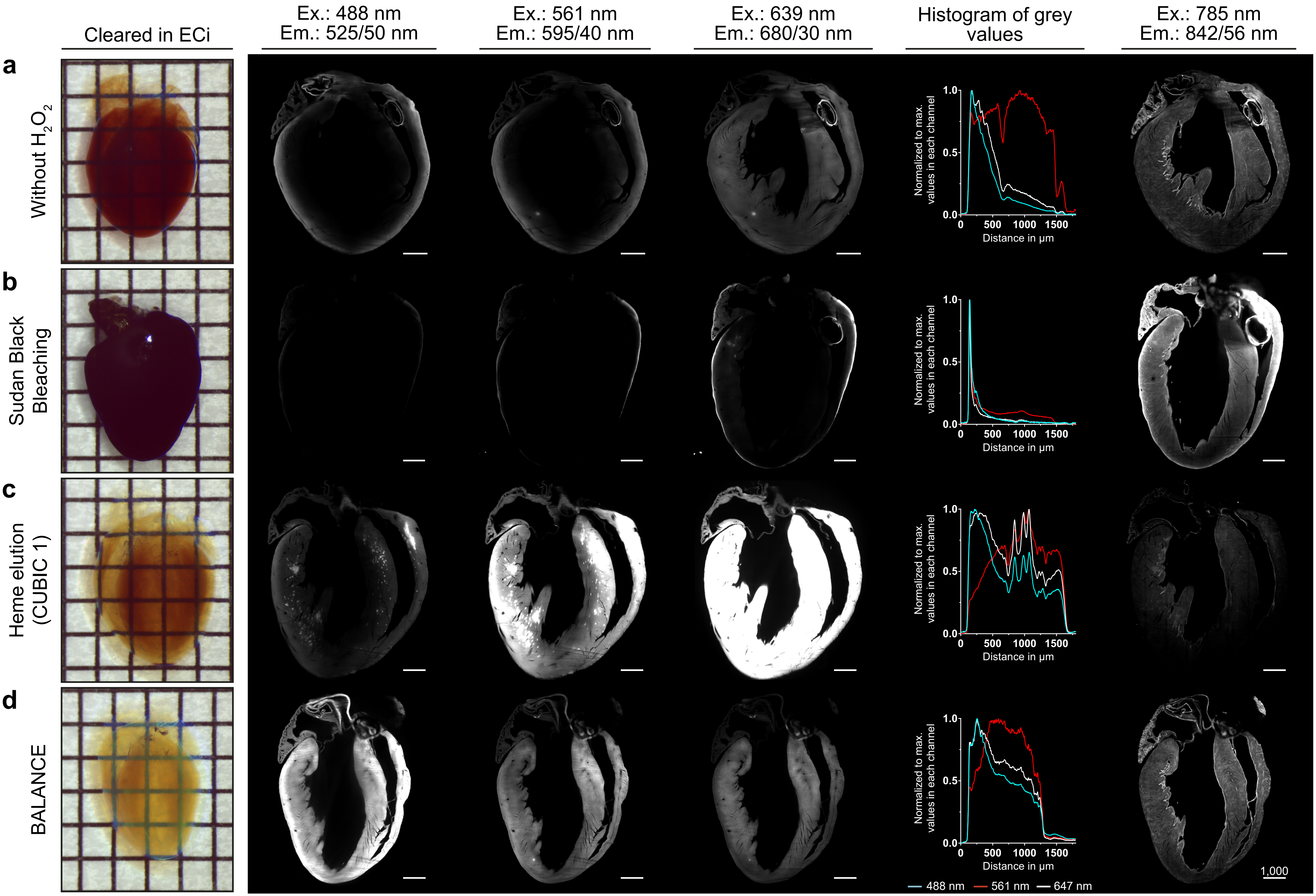

Supplementary Figure 1 Tissue autofluorescence homogenization using different bleaching protocols. Evaluation of ECi clearing combined with (**a**) no bleaching[3], (**b**) Sudan Black bleaching[1], (**c**) heme elution (CUBIC 1)[1,2] and (**d**) peroxide (H_2O_2)-based bleaching (BALANCE) with regard to signal tissue penetration in indicated fluorescent channels. Scale bar values in µm. One square in the macroscopic images is 2x2 mm. Source data are provided as a Source Data file. (ECi – ethyl cinnamate, Ex. – excitation, Em. – emission)

Supplementary Figure 2

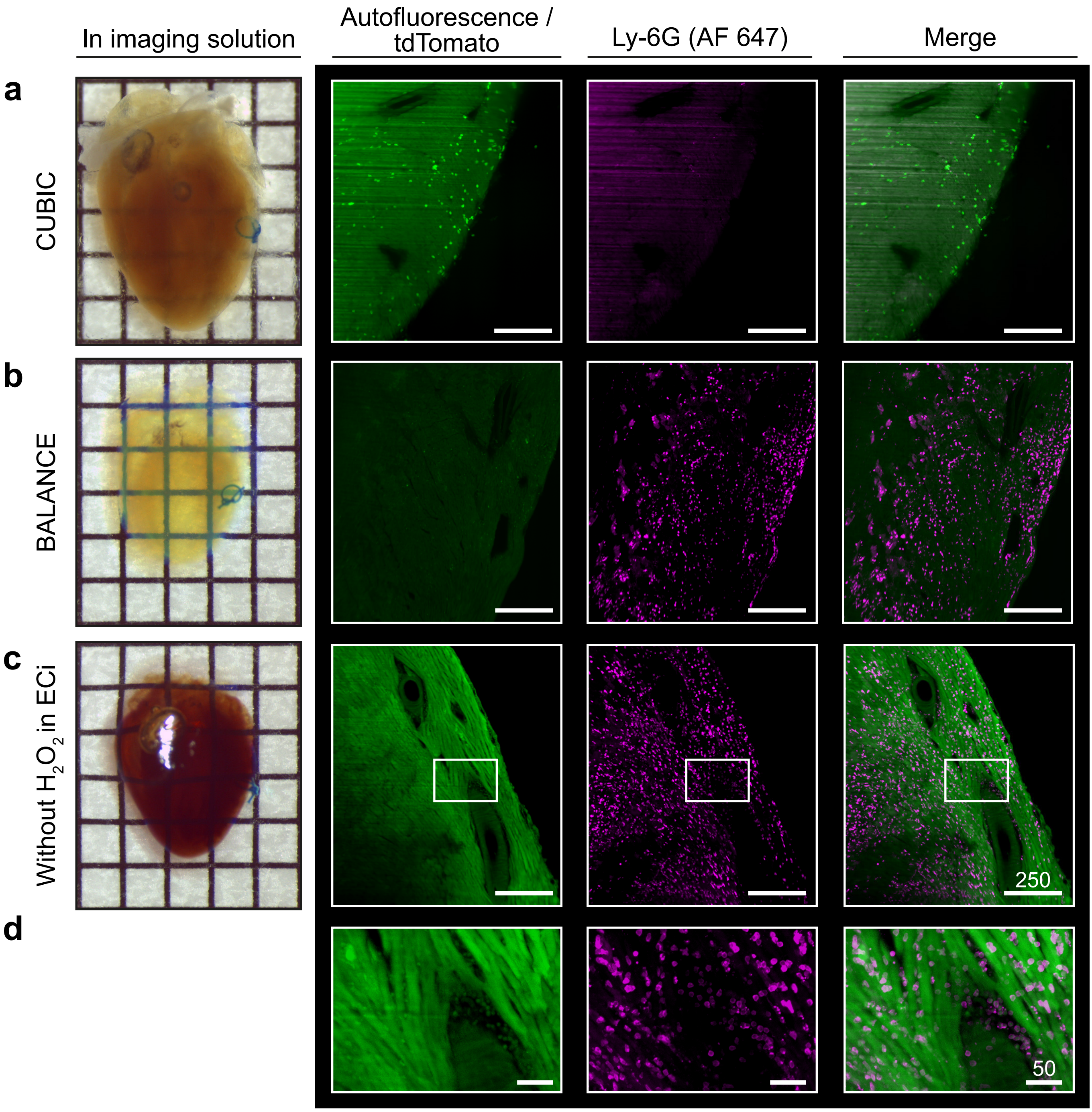

Supplementary Figure 2 Endogenous fluorescence and i.v.-mediated staining in organic- and water-based clearing. Comparison of (**a**) CUBIC[5], (**b**) BALANCE and (**c**) ECi-only[3] clearing in their capabilities of preserving endogenous (tdTomato, green) and artificial fluorophore (anti-mLy-6G AF647, magenta) fluorescence in Catchup mouse[4] hearts (neutrophils tdTomato positive) after myocardial ischemia/reperfusion (I/R) injury. (**d**) Magnification of ROIs depicted in c with white rectangles. Scale bar values in µm. One square on the macroscopic images (left) equals 2x2 mm.

Supplementary Figure 3

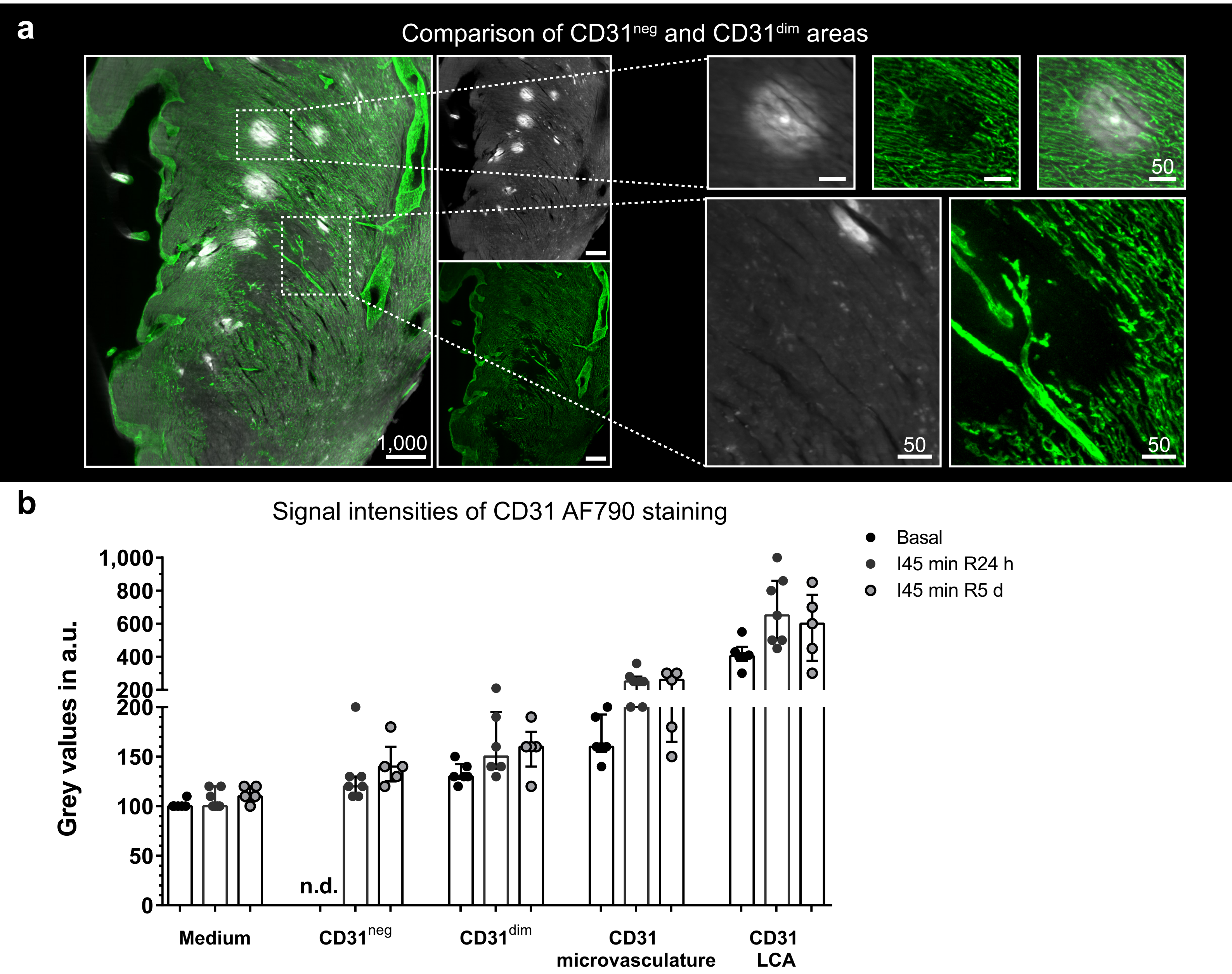

Supplementary Figure 3 CD31 imaging in the heart. (**a**) *Left:* ROI of an exemplary infarcted heart at 8x magnification showing CD31 (green) and autofluorescence (grey). *Right:* ROIs depicted on the left side in digital magnification. On the top, co-localization of a CD31 dim ($CD31^{dim}$) area (green) and high autofluorescence (grey to white) is shown. At the bottom, CD31 negative ($CD31^{neg}$) areas do not co-localize with increased autofluorescence. (**b**) CD31-AlexaFluor 790 raw values from various experimental end points and heart structures as indicated (median ± interquartile range; n=5). Scale bar values in µm. Source data are provided as a Source Data file.

Supplementary Figure 4

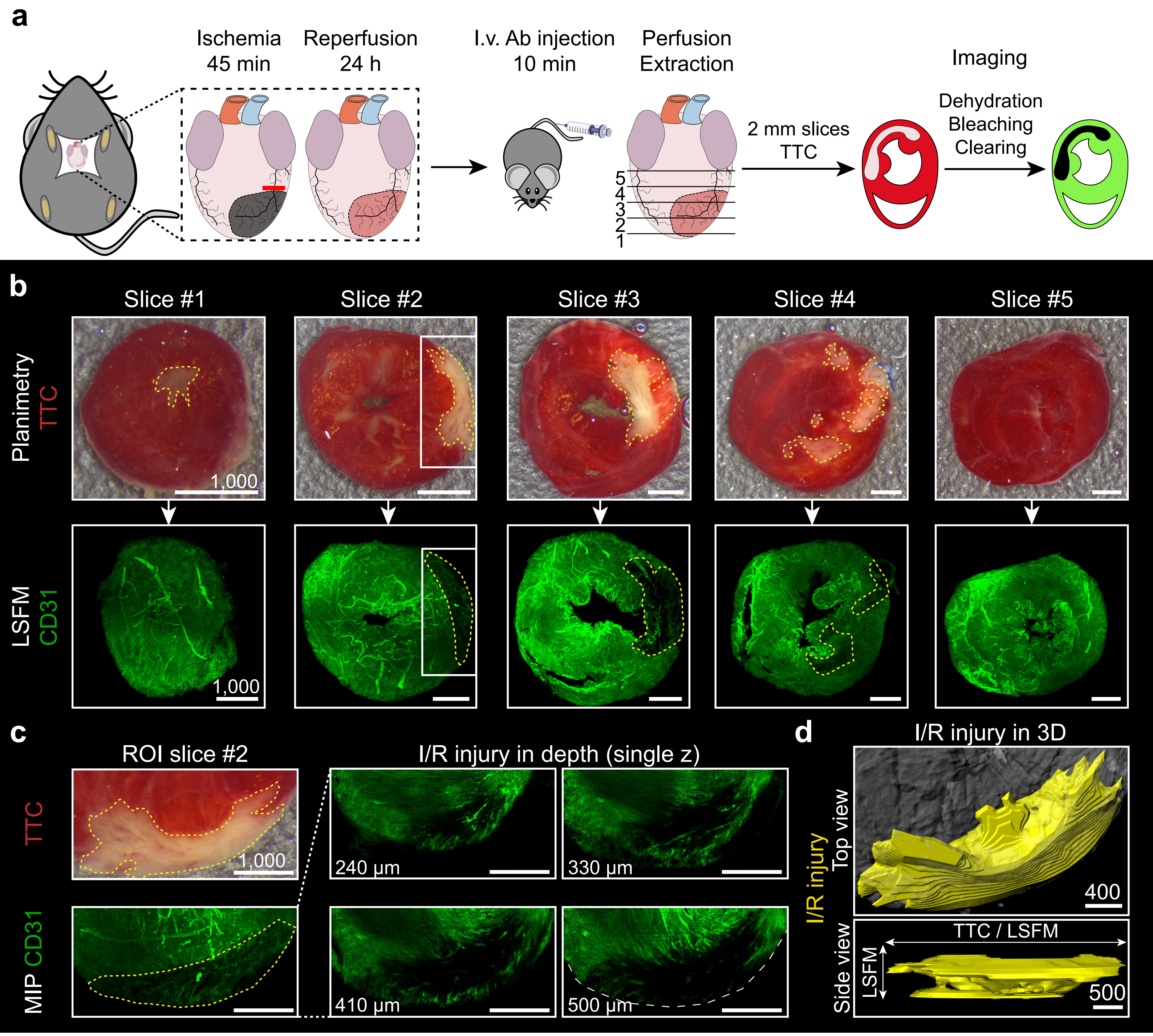

Supplementary Figure 4 TTC-planimetry and subsequent LSFM of murine hearts in a myocardial ischemia/reperfusion (I/R) model. (**a**) Workflow for comparison of triphenyl tetrazolium chloride (TTC)- and LSFM-based analysis in single heart slices. (**b**) Slice by slice comparison of an exemplary heart analyzed first by TTC-planimetry (upper row), followed by LSFM (maximum intensity projections (MIP), lower row) of the same slice (white = TTC negative / metabolically inactive, red = TTC positive / metabolically active). TTC negative / CD31 negative areas (dashed yellow lines) are largely overlapping. (**c**) *Left:* Comparison of TTC and LSFM-MIP from slice #2 in (b). *Right:* single optical slices in z depth, indicating complete loss of CD31 signal in a 3D structure. (**d**) 3D volume visualization of CD31 negative signal from the slice depicted in (c). (**e**) Correlation of TTC negative and CD31 negative area sizes in the same slice (r- and p-values from linear regression given, n=3 hearts, 5 slices per heart). Scale bar values in µm.

Supplementary Figure 5

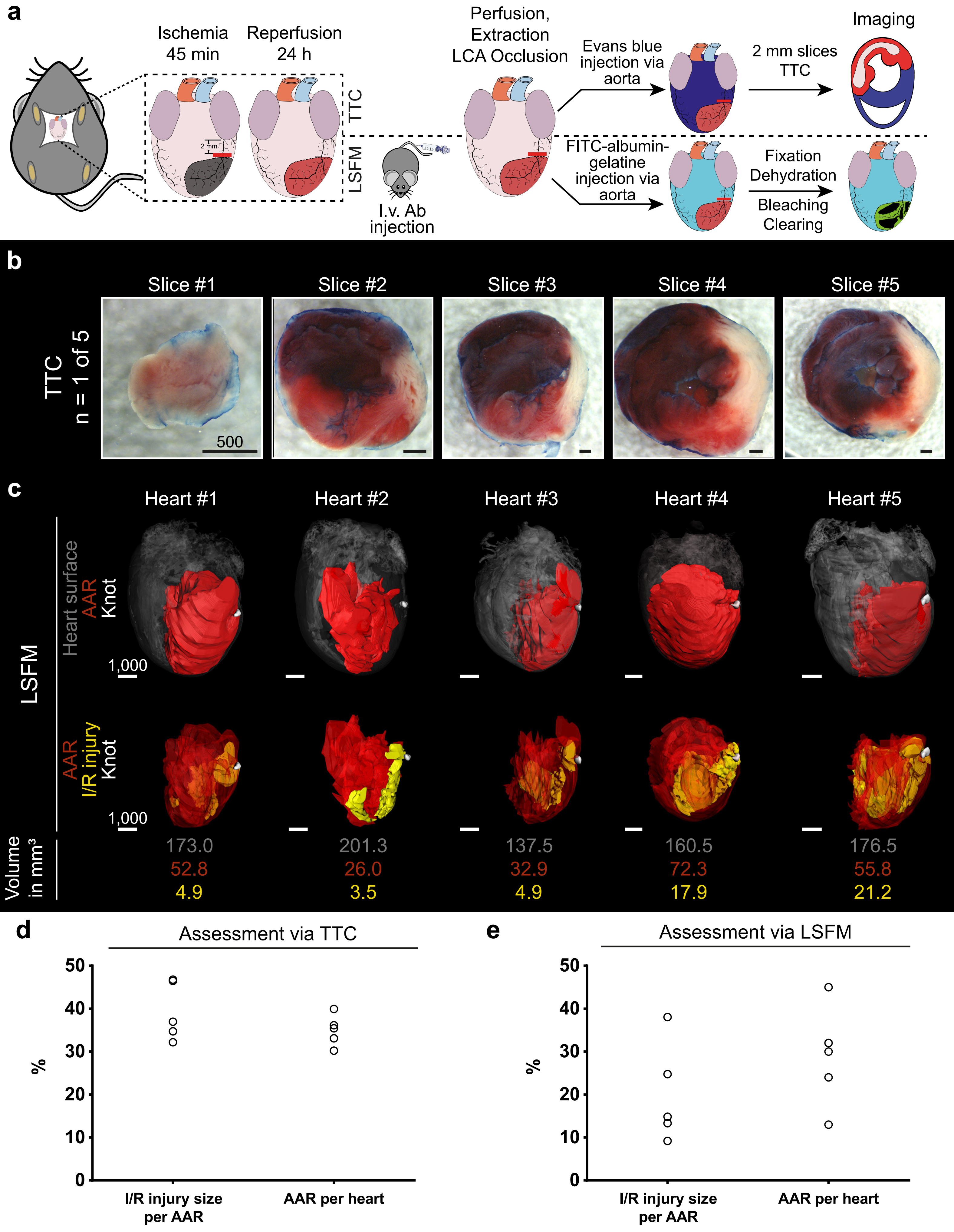

Supplementary Figure 5 Ischemia/reperfusion (I/R) injury parameters in 3D – triphenyl tetrazolium chloride (TTC)- and light sheet fluorescence microscopy (LSFM)-based analysis in comparison. (**a**) Workflow for 2D TTC- and 3D LSFM-based analysis of myocardial I/R injury. (**b**) Planimetry of 2 mm thick slices of one exemplary heart stained with TTC / Evans blue (EB; White = TTC negative / metabolically inactive, red = TTC positive / metabolically active, not blue = EB negative = area at risk (AAR)). (**c**) Five hearts were analyzed using LSFM and in silico post-processing. All hearts are shown with their anterior side to the front, the knot (white) being on the right side. Upper row*:* heart surface (translucent grey) together with AAR volume (red). Lower row*:* same hearts with traced I/R injury volume (yellow). Numbers at the bottom represent heart (grey), AAR (red) and I/R injury volume (yellow) for the respective heart. (**d**) I/R injury size per AAR and AAR per heart as assessed by TTC and EB staining (n=5). (**e**) I/R injury size per AAR and AAR per heart as assessed by CD31 and FITC staining (n=5). Hearts depicted in (d) and (e) stem from separate cohorts. Scale bar values in µm. Source data are provided as a Source Data file.

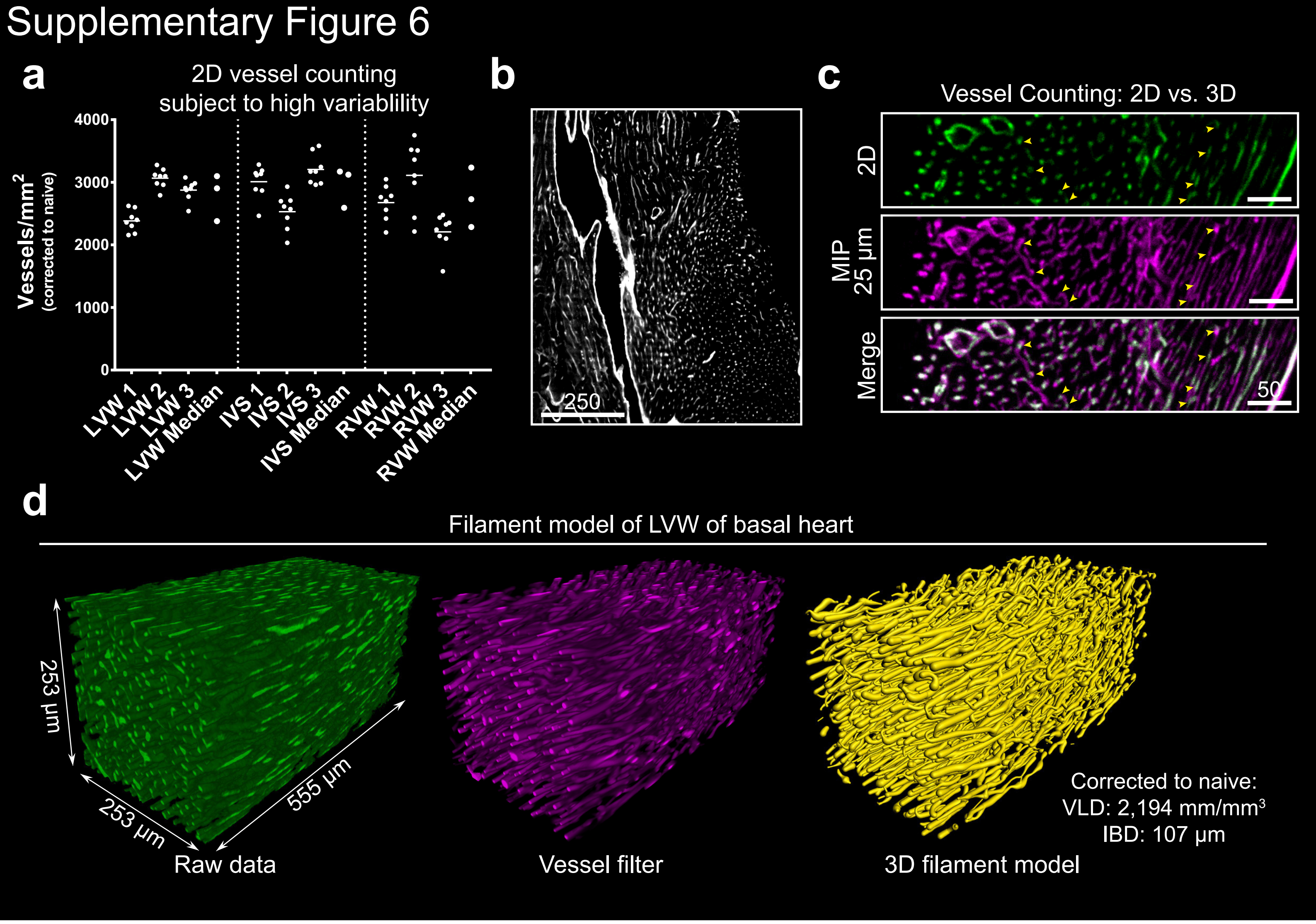

Supplementary Figure 6 2D and 3D cardiac vessel measurements. (**a**) Quantification of CD31 positive vessels in optical slices at different z positions of the right ventricular wall (RVW), intraventricular septum (IVS) and left ventricular wall (LVW) of the murine heart (n=3 mice, each dot represents one counted ROI; the respective median, as well as a median summary for each compartment is shown; values have been corrected for shrinkage). (**b**) Exemplary field of view showing diverse vessel directionality in LVW. (**c**) Images show that counting in 2D (e.g. optical slice) is difficult and results in repeated measures of the same vessel (yellow arrows) visualized by a maximum intensity projection (MIP) of 25 µm z depth (magenta). Magnification: 6.4x. (**d**) Computing a 3D filament model: from a 3D reconstruction of the raw data (green) via a vessel-filtered data set (magenta) using the FRANGI algorithm to the 3D filament model (yellow) of the LVW microvasculature. This model allows in-depth analysis of the vascular network, including parameters like vessel length density (VLD) and measures of complexity e.g. interbranch distance (IBD). Based on a magnification of 6.4x. This represents one experiment (n=1). Scale bar values in µm. Source data are provided as a Source Data file.

Supplementary Figure 7

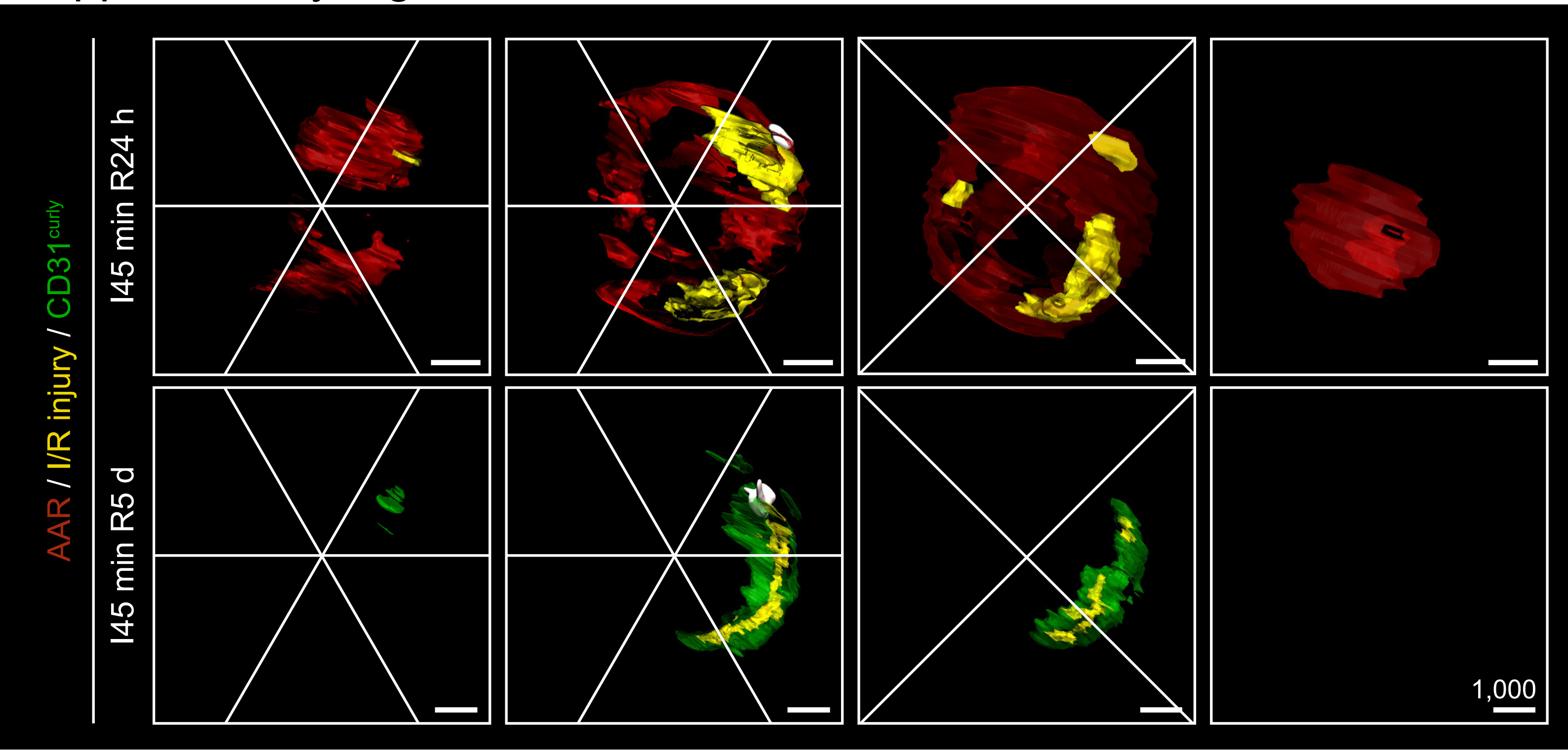

Supplementary Figure 7 Quantifying ischemia/reperfusion (I/R) injury, area at risk (AAR) and CD31 curly localization. To quantify the detailed localization of I/R injury, AAR and CD31curly, we used the left ventricular 17 segment model[7] in mice in combination with 3D LSFM data. Representative examples of scored hearts after 24 h (upper row) and 5 d (lower row) of reperfusion depicting AAR (red), CD31 negative (yellow) and newly vascularized CD31 curly (green) volumes are shown. Scale bar values in µm.

Supplementary Figure 8

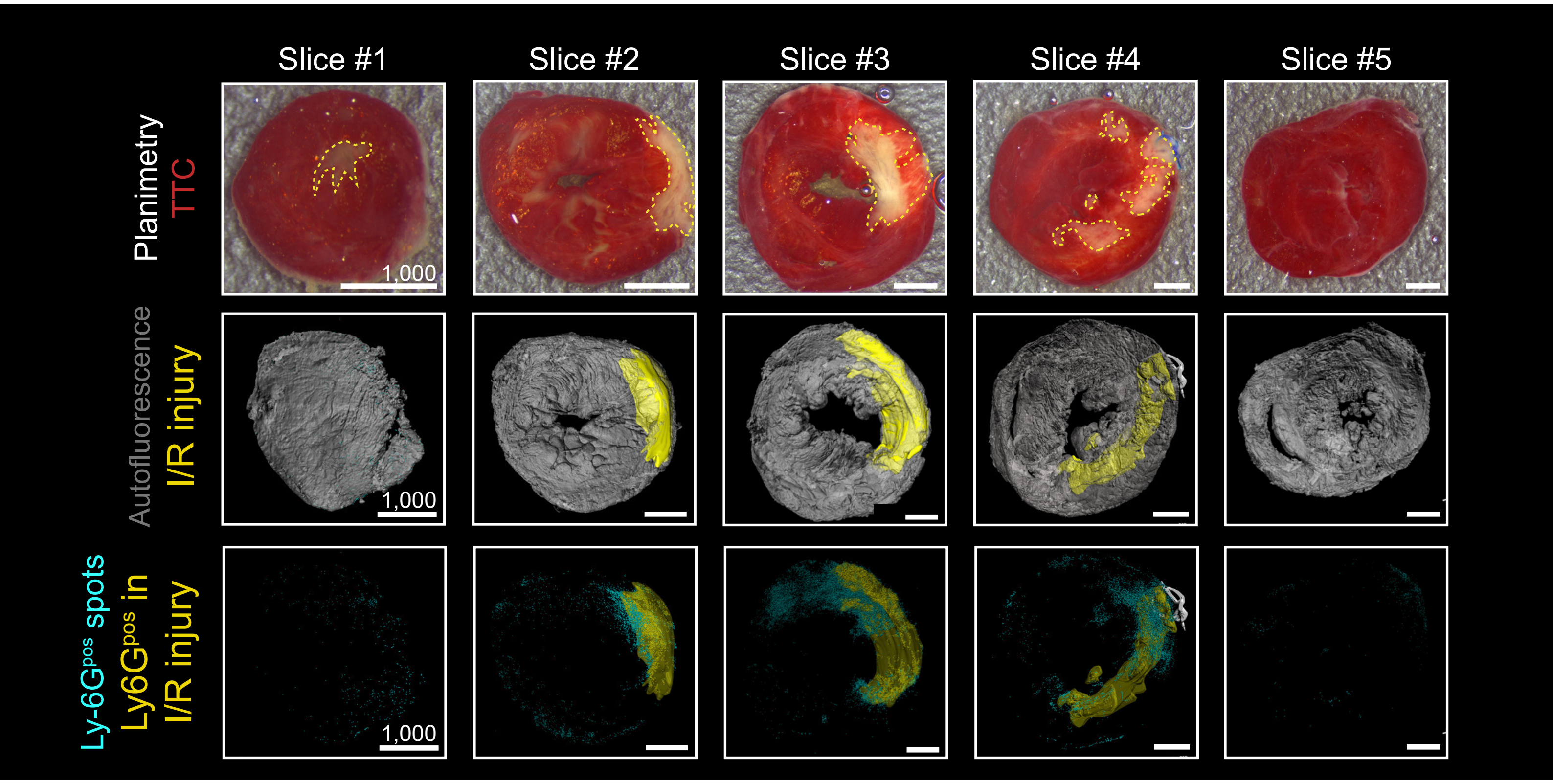

Supplementary Figure 8 Co-localization of ischemia/reperfusion (I/R) injury with Ly-6G positive (Ly-6G^{pos}) neutrophils. *Top:* 2 mm thick tetrazolium chloride (TTC) slices as already shown in Supplementary Figure 4 for orientation. *Middle:* 3D reconstruction of slices showing autofluorescence (grey) and CD31 negative (CD31^{neg})/ I/R injury volume (yellow). *Bottom:* Total Ly-6G^{pos} neutrophils (turquoise) were detected using IMARIS spot function. Ly-6G^{pos} spots inside the I/R injury are colored in yellow. These findings were verified in 3 mice. Scale bar values in µm.

Supplementary Figure 9

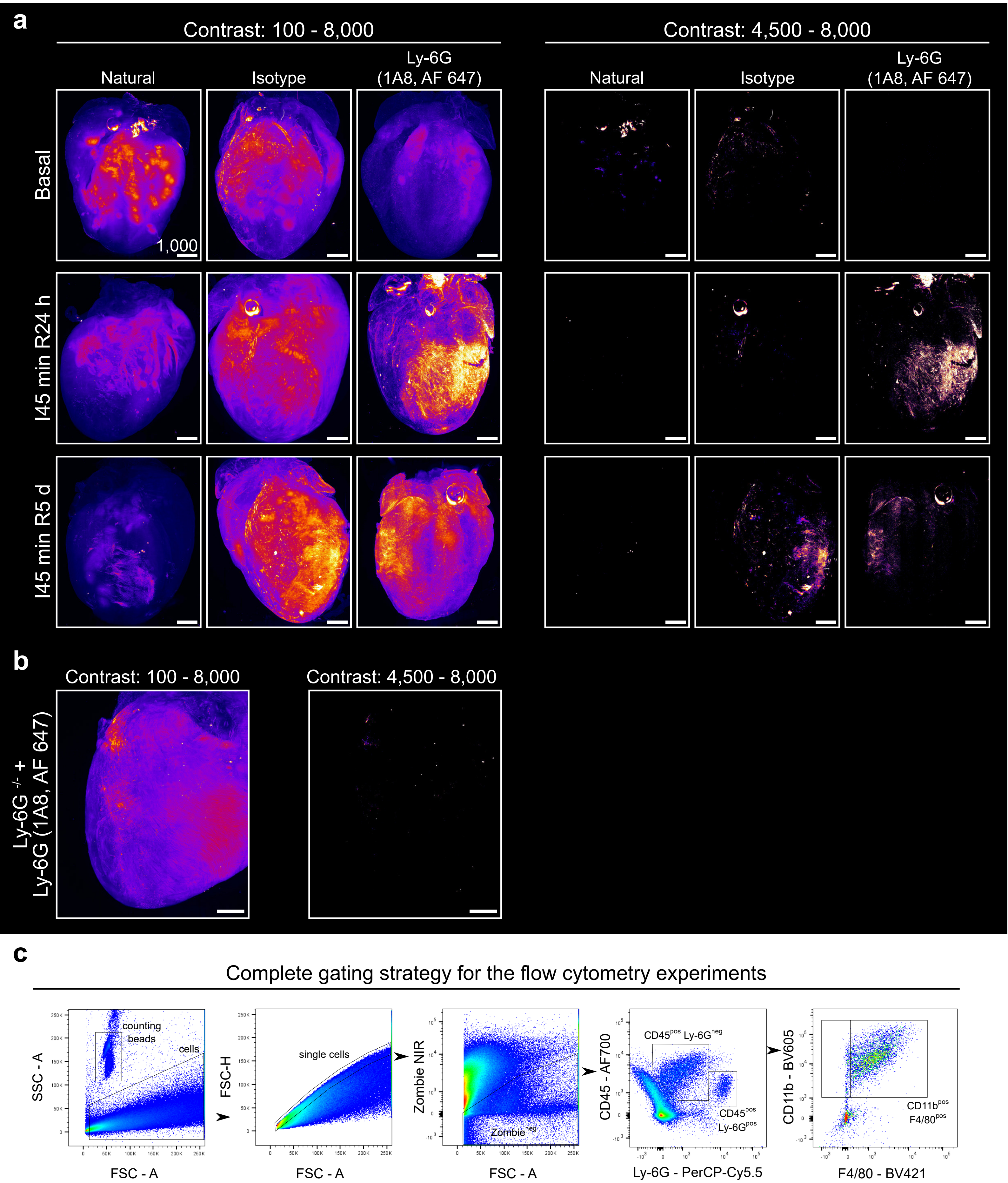

Supplementary Figure 9 Immune cell identification in LSFM and flow cytometry analysis. (**a**) *Left:* Ly-6G AlexaFluor 647 signal obtained from various, exemplary hearts displayed as a heat map of greyscale values ranging from 100 to 8000 counts. *Right:* same hearts as depicted on the left, with a narrower greyscale value (4500 – 8000 counts), adjusted to minimize signal in the baseline heart with Ly-6G AlexaFluor 647 antibody (right top image). (**b**) As a control, Ly-6G$^{-/-}$ knockout mice were injected with Ly-6G AlexaFluor 647 antibody 24 h after ischemia and processed and imaged as before. Ly-6G positive (Ly-6G^{pos}) signals inside the heart could be obtained with a wide threshold (100 – 8000 counts greyscale value), but were no longer detectable with the narrow threshold applied above (4500 – 8000 counts greyscale value). (**c**) Flow cytometry gating strategy. Pseudo color density plots of an exemplary heart analyzed by flow cytometry are shown. After gating for forward and side scatter, doublet cells and dead/alive staining, cells are gated for CD45 and Ly-6G expression and further analyzed as displayed. CD45^{pos} / Ly-6G^{pos} cells are considered neutrophils. CD45^{pos} / Ly-6G^{neg} cells are gated for CD11b and F4/80 expression. CD11b^{pos} / F4/80^{pos} cells are considered macrophages. Total cell numbers per heart are obtained using counting beads.

Supplementary References

1 Treweek, J. B. *et al.* Whole-body tissue stabilization and selective extractions via tissue-hydrogel hybrids for high-resolution intact circuit mapping and phenotyping. *Nat Protoc* **10**, 1860-1896, doi:10.1038/nprot.2015.122 (2015).

2 Susaki, E. A. *et al.* Advanced CUBIC protocols for whole-brain and whole-body clearing and imaging. *Nat Protoc* **10**, 1709-1727, doi:10.1038/nprot.2015.085 (2015).

3 Klingberg, A. *et al.* Fully Automated Evaluation of Total Glomerular Number and Capillary Tuft Size in Nephritic Kidneys Using Lightsheet Microscopy. *J Am Soc Nephrol* **28**, 452-459, doi:10.1681/ASN.2016020232 (2017).

4 Hasenberg, A. *et al.* Catchup: a mouse model for imaging-based tracking and modulation of neutrophil granulocytes. *Nat Methods* **12**, 445-452, doi:10.1038/nmeth.3322 (2015).

5 Orlich, M. & Kiefer, F. A qualitative comparison of ten tissue clearing techniques. *Histol Histopathol* **33**, 181-199, doi:10.14670/HH-11-903 (2018).

6 Yu, T., Qi, Y., Gong, H., Luo, Q. & Zhu, D. Optical clearing for multiscale biological tissues. *J Biophotonics* **11**, doi:10.1002/jbio.201700187 (2018).

7 Cerqueira, M. D. *et al.* Standardized myocardial segmentation and nomenclature for tomographic imaging of the heart. A statement for healthcare professionals from the Cardiac Imaging Committee of the Council on Clinical Cardiology of the American Heart Association. *Circulation* **105**, 539-542 (2002).
