# Supplementary material for: Contemporaneous 3D characterization of acute and chronic myocardial I/R injury and response
Source: Nat Commun. 2019 May 24;10:2312. doi: 10.1038/s41467-019-10338-2 (PMC6534576; doi:10.1038/s41467-019-10338-2)
Supplement: Supplementary file 3 — Description of Additional Supplementary Files [file 41467_2019_10338_MOESM3_ESM.docx]

**Description of Additional Supplementary Files**

**File Name: Supplementary Movie 1**

**Description:** 3D structure of the aortic valve. 3D LSFM reconstruction of the aortic valve, mostly shown from the top looking inside the left ventricle, based on CD31 (green) and autofluorescence (grey) signal. The three leaflets of the valve and the beginning of the coronary arteries directly above the valve can be seen.

**File Name: Supplementary Movie 2**

**Description:** Determination of the area at risk (AAR) in 3D. The video shows, how AAR volume is reconstructed in 3D based on a fluorescein isothyocyanate (FITC)- albumin/gelatin filling of an I45minR24h murine heart and semi-/manual tracing. These findings were verified in at least 5 mice.

**File Name: Supplementary Movie 3**

**Description:** Determination of I/R injury in 3D. The video shows, how I/R injury volume is reconstructed in 3D based on semiautomatic tracing of CD31 negative (CD31neg) regions, here in an I45minR24h murine heart. This can be compared to the previously traced area at risk (AAR) (Supplementary Movie 2) in the same heart. These findings were verified in at least 5 mice.

**File Name: Supplementary Movie 4**

**Description:** Interplay of traced vessels with I/R injury parameters. Here, major vessels of an I45minR24h murine heart were traced. Discrimination of arteries and veins was done based on the vessels’ respective location within the muscle (veins on the outside, arteries within the tissue) and on their CD31 signal intensity (CD31high = arteries, CD31low = veins). Taking the position of the knot into account, determination of the occluded artery branches is possible. This vessel-based information of the area at risk (AAR) matches the volume traced before using the FITC-albumin filling as a guide. Interestingly, only parts of the microvasculature lining the major vessels traced show CD31 signal absence as expressed by the traced I/R volume. These findings were verified in at least 5 mice.

**File Name: Supplementary Movie 5**

**Description:** Ly-6G positive (Ly-6Gpos) cells accumulate at the border of CD31 negative (CD31neg) areas. In this 3D rendering of an I45minR24h heart, the interplay of CD31 (green) negative regions (I/R injury) and Ly-6Gpos (turquoise) neutrophils is depicted. These findings were verified in at least 5 mice.

**File Name: Supplementary Movie 6**

**Description:** CD31 curly (CD31curly) regions are penetrated by Ly-6G positive (Ly-6Gpos) cells. In this 3D rendering of an I45minR5d heart, CD31curly, I/R injury and Ly6Gpos neutrophils (turquoise) are depicted in contrast to Supplementary Movie 5. These findings were verified in at least 5 mice.

**File Name: Supplementary Movie 7**

**Description:** Neutrophil localization in respect to I/R injury parameters. Here, we colored the Ly-6G positive (Ly6Gpos) neutrophil spots in respect to their precise location after 24 h of reperfusion: within I/R injury (yellow), within the area at risk (AAR, red) and further away (turquoise), depicted in the same heart as in Supplementary Movie 5. These findings were verified in at least 5 mice.

**File Name: Supplementary Movie 8**

**Description:** Neutrophil localization in respect to I/R injury parameters. Here, we colored the Ly-6G positive (Ly-6Gpos) neutrophil spots in respect to their precise location after 5 d of reperfusion: within I/R injury (yellow), within the CD31 curly region (CD31curly, green) and further away (turquoise). Video shows the same heart as in Supplementary Movie 6. These findings were verified in at least 5 mice.
