# Supplementary material for: Contemporaneous 3D characterization of acute and chronic myocardial I/R injury and response
Source: Nat Commun. 2019 May 24;10:2312. doi: 10.1038/s41467-019-10338-2 (PMC6534576; doi:10.1038/s41467-019-10338-2)
Supplement: Supplementary file 12 — Reporting summary [file 41467_2019_10338_MOESM12_ESM.pdf]

nature research

Corresponding author(s): Matthias Totzeck
Last updated by author(s): May 7, 2019

Reporting Summary

Nature Research wishes to improve the reproducibility of the work that we publish. This form provides structure for consistency and transparency in reporting. For further information on Nature Research policies, see Authors & Referees and the Editorial Policy Checklist.

Statistics

For all statistical analyses, confirm that the following items are present in the figure legend, table legend, main text, or Methods section.

n/a	Confirmed	
☐	☒	The exact sample size (*n*) for each experimental group/condition, given as a discrete number and unit of measurement
☐	☒	A statement on whether measurements were taken from distinct samples or whether the same sample was measured repeatedly
☐	☒	The statistical test(s) used AND whether they are one- or two-sided<br>*Only common tests should be described solely by name; describe more complex techniques in the Methods section.*
☐	☒	A description of all covariates tested
☐	☒	A description of any assumptions or corrections, such as tests of normality and adjustment for multiple comparisons
☐	☒	A full description of the statistical parameters including central tendency (e.g. means) or other basic estimates (e.g. regression coefficient) AND variation (e.g. standard deviation) or associated estimates of uncertainty (e.g. confidence intervals)
☐	☒	For null hypothesis testing, the test statistic (e.g. *F*, *t*, *r*) with confidence intervals, effect sizes, degrees of freedom and *P* value noted<br>*Give P values as exact values whenever suitable.*
☒	☐	For Bayesian analysis, information on the choice of priors and Markov chain Monte Carlo settings
☒	☐	For hierarchical and complex designs, identification of the appropriate level for tests and full reporting of outcomes
☐	☒	Estimates of effect sizes (e.g. Cohen's *d*, Pearson's *r*), indicating how they were calculated

Our web collection on statistics for biologists contains articles on many of the points above.

Software and code

Policy information about availability of computer code

Data collection	BD Diva; Leica Application Suite v4.5; ImspectorPro_5_0_285; Imaris v.9.2
Data analysis	FIJI/ImageJ; FlowJo v10; ImarisFileConverterx64 v9.2; Imaris v.9.2; R v3.4.4; GraphPad Prism v6

For manuscripts utilizing custom algorithms or software that are central to the research but not yet described in published literature, software must be made available to editors/reviewers. We strongly encourage code deposition in a community repository (e.g. GitHub). See the Nature Research guidelines for submitting code & software for further information.

Data

Policy information about availability of data

All manuscripts must include a data availability statement. This statement should provide the following information, where applicable:

- Accession codes, unique identifiers, or web links for publicly available datasets
- A list of figures that have associated raw data
- A description of any restrictions on data availability

The datasets generated during and/or analyzed during the current study are available from the corresponding authors upon request. The source data underlying figures 1, 4, 5-7, and supplementary figure 1, 3, 5 and 6 are provided as a Source Data file.

Field-specific reporting

Please select the one below that is the best fit for your research. If you are not sure, read the appropriate sections before making your selection.

☒ Life sciences ☐ Behavioural & social sciences ☐ Ecological, evolutionary & environmental sciences

Life sciences study design

All studies must disclose on these points even when the disclosure is negative.

Sample size	This is an exploratory examination. Sample size was planned for experiments using in vivo I/R injury as previously published by us (Hendgen-Cotta et al Proc Natl Acad Soc 2008; Luedike et al. Circ 2012, Rassaf et al. Circ. Res. 2014, Michel et al J Vis Exp 2018, Totzeck et al. J Biol Methods 2016).
Data exclusions	Data was excluded based on mice physical appearance before and after operation until the end of the experiment.
Replication	Experiments were conducted in replicate as outlined in the manuscript in detail. All corresponding data are available in the manuscript.
Randomization	Mice were allocated randomly to basal, sham or ischemia/reperfusion group as well as the different reperfusion end points.
Blinding	Mice operators were blinded to reperfusion times and data collection and analyzing operators were blinded to the operation procedure (basal, sham or ischemia/reperfusion; 24 h or 5 d of reperfusion).

Reporting for specific materials, systems and methods

We require information from authors about some types of materials, experimental systems and methods used in many studies. Here, indicate whether each material, system or method listed is relevant to your study. If you are not sure if a list item applies to your research, read the appropriate section before selecting a response.

Materials & experimental systems

n/a	Involved in the study	
☐	☒	Antibodies
☒	☐	Eukaryotic cell lines
☒	☐	Palaeontology
☐	☒	Animals and other organisms
☐	☒	Human research participants
☒	☐	Clinical data

Methods

n/a	Involved in the study	
☒	☐	ChIP-seq
☐	☒	Flow cytometry
☒	☐	MRI-based neuroimaging

Antibodies

Antibodies used	anti-mouse CD31 (clone: Mec13.3; purified: CatNo. 553369, BD; conjugated with AlexaFluor (AF) 647: CatNo. 102516 BioLegend), Ly-6G (clone: 1A8; purified: CatNo. 127602, BioLegend; conjugated with AF647: CatNo. 127610, BioLegend), F4/80 antibody (clone: BM8; purified: CatNo. 123102, BioLegend; conjugated with AF594: CatNo. 123140, BioLegend), CD45-AF700 (CatNo 103127, BioLegend), Ly-6G-PerCP/Cy5.5 (CatNo 127615, BioLegend), CD11b-BV605 (CatNo 101257, BioLegend) and F4/80-BV421 (CatNo 123137, BioLegend)
Validation	Validation of primary antidbodies as per manufacturer's statement:<br>• anti-mouse CD31 (clone: Mec13.3; purified: CatNo. 553369, BD): The MEC13.3 antibody specifically recognizes CD31, also known as PECAM-1 (Platelet Endothelial Cell Adhesion Molecule-1)<br>• anti-mouse CD31 (clone: Mec13.3; conjugated with AlexaFluor (AF) 647: CatNo. 102516 BioLegend): Anti-mouse CD31 clones 390 and MEC13.3 bind to their respective non-overlapping epitopes in IgD2 of CD31. Additional reported applications (in the relevant formats) include: immunoprecipitation1, in vitro and in vivo blocking of CD31-mediated cell-cell interactions, and immunohistochemical staining of acetone-fixed frozen sections and zinc-fixed paraffin-embedded sections.<br>• Ly-6G (clone: 1A8; purified: CatNo. 127602, BioLegend): FC - Quality tested; IHC - Reported in the literature; Additional reported applications (for the relevant formats) include: immunohistochemistry of frozen sections and paraffin-embedded sections, and depletion.<br>• Ly-6G (clone: 1A8;conjugated with AF647: CatNo. 127610, BioLegend): FC - Quality tested; IHC-F – Validated; Additional reported applications (for the relevant formats) include: immunohistochemistry of frozen sections and paraffin-embedded sections, and depletion.<br>• F4/80 antibody (clone: BM8; purified: CatNo. 123102, BioLegend): FC - Quality tested; IHC, WB - Reported in the literature; Additional reported applications (for the relevant formats) include: immunohistochemical staining of acetone-fixed frozen section and formalin-fixed paraffin-embedded sections, and Western blotting.<br>• F4/80 antibody (clone: BM8; conjugated with AF594: CatNo. 123140, BioLegend): IHC, IF - Quality tested; Additional reported applications (for the relevant formats) include: immunohistochemical staining of acetone-fixed frozen sections and formalin-fixed paraffin-embedded sections, and Western blotting.<br>• CD45-AF700 (CatNo 103127, BioLegend): FC - Quality tested<br>• Ly-6G-PerCP/Cy5.5 (CatNo 127615, BioLegend): FC - Quality tested<br>• CD11b-BV605 (CatNo 101257, BioLegend): FC - Quality tested<br>• F4/80-BV421 (CatNo 123137, BioLegend): FC - Quality tested; IHC-F - Validated

Animals and other organisms

Policy information about studies involving animals; ARRIVE guidelines recommended for reporting animal research

Laboratory animals	C57BL/6JRJ and Catchup (C57BL/6-Ly6g(tm2621(Cre-tdTomato)Arte) mice, male, 9-15 weeks of age
Wild animals	The study did not involve wild animals
Field-collected samples	The study did not involve samples collected from the field
Ethics oversight	All experimental animal procedures were approved by the responsible governmental agency, the Ministry for Environment, Agriculture, Conservation and Consumer Protection of the State of North Rhine-Westphalia (MULNV) and complied with all relevant ethical regulations for animal testing and research.

Note that full information on the approval of the study protocol must also be provided in the manuscript.

Human research participants

Policy information about studies involving human research participants

Population characteristics	Patients undergoing coronary bypass surgery
Recruitment	Human left atrial appendage (LAA) biopsies were routinely excised during coronary bypass surgery presenting no further harm for the patient. After anonymization, samples were processed.
Ethics oversight	All human studies were approved by the local ethics committee of the Medical Faculty, University of Duisburg-Essen, Germany (Ethics Approval Nr. 18-8527-BO).The whole procedure was in accordance to the World Medical Association Declaration of Helsinki and conducted in accordance with the relevant ethical regulations. Written informed consent was obtained from all participants. The patients underwent coronary bypass surgery, during which human left atrial appendage (LAA) biopsies were routinely excised. This presented no further harm for the patient.

Note that full information on the approval of the study protocol must also be provided in the manuscript.

Flow Cytometry

Plots

Confirm that:

☒ The axis labels state the marker and fluorochrome used (e.g. CD4-FITC).

☒ The axis scales are clearly visible. Include numbers along axes only for bottom left plot of group (a 'group' is an analysis of identical markers).

☒ All plots are contour plots with outliers or pseudocolor plots.

☒ A numerical value for number of cells or percentage (with statistics) is provided.

Methodology

Sample preparation	PBS-perfused hearts of mice after 24 h and 5 d of reperfusion as well as under basal conditions, were mechanically minced and incubated in an enzyme solution comprised of 450 U/ml collagenase I (CatNo C0130, Sigma Aldrich), 125 U/ml collagenase XI (CatNo C7657, Sigma Aldrich), 60 U/ml hyaluronidase (CatNo H3506, Sigma Aldrich), 20mM HEPES (CatNo H3375, Sigma Aldrich), 60 U/ml DNase (CatNo D5319, Sigma Aldrich) in PBS for 40 min in a ThermoMixer C (Eppendorf) at 37 °C and 300 rpm. After enzymatic digestion, the solution was filtered through a 40 µm filter and flow-through was centrifuged for 5 min at 4 °C and 350x g. Supernatant was discarded and cell pellet resuspended in 1.5 ml PBS. 100 µl of this solution per sample was used for staining, while one solution was chosen for "fluorescence minus one" (FMO) controls. Blocking of FC receptors was done by adding 2 µl of TruStain fcX (CatNo 101319, BioLegend) and incubating for 20 min on ice in the dark. Samples were washed by adding 100 µl PBS, centrifuging for 5 min at 4 °C and 350x g, discarding supernatant, and resuspending the cell pellet in 50 µl PBS. Samples were stained by adding 50 µl PBS containing antibodies CD45-AF700 (CatNo 103127, BioLegend), Ly-6G-PerCP/Cy5.5 (CatNo 127615, BioLegend), CD11b-BV605 (CatNo 101257, BioLegend) and F4/80-BV421 (CatNo 123137, BioLegend) diluted 1:200. Incubation was done for 30 min at RT in the dark, with simultaneous dead and alive staining by addition of Zombie NIR dye (1:2000, CatNo 423105, BioLegend). After antibody incubation, samples were washed as before, supernatant was discarded and cell pellet was resuspended in 150 µl FACS-Buffer, comprised of 1 % (v/v) fetal bovine serum (CatNo P30-3302, PAN Biotech) and 0.5 % bovine serum albumin (CatNo 8076.3, Roth) in PBS.
Instrument	BD FACSAria III
Software	For data collection, BD Diva software was used. For data analysis, we employed FlowJo software.
Cell population abundance	Cells were not sorted after flow cytometry and cell population purity was determined by fluorescence minus one (FMO) controls.
Gating strategy	Cells were gated first for FSC/SSC, doublets and alive cells using Zombie near-infrared (NIR) dye. Then CD45-AF700 positive/ Ly-6G-PerCP-Cy5.5 positive cells were determined neutrophils. From the CD45-AF700 positive/Ly-6G-PerCP-Cy5.5 negative cells,

expression of CD11b-BrilliantViolet605 and F4/80-BrilliantViolet421 was determined and double positive cells were labeled macrophages. Zombie NIR, CD45-AF700, Ly-6G-PerCP-Cy5.5, CD11b-BrilliantViolet605 and F4/80-BrilliantViolet421 populations were defined using fluorescence-minus-one controls.

☒ Tick this box to confirm that a figure exemplifying the gating strategy is provided in the Supplementary Information.
